# Identification of the nucleolar localization signal in Autographa californica multiple nucleopolyhedrovirus multifunctional protein Ac16

**DOI:** 10.1099/jgv.0.002246

**Published:** 2026-04-01

**Authors:** Guoqing Chen, Yihong Wu, Jing Yang, Haoran Wang, Xinxin Zhang, Guozhong Feng

**Affiliations:** 1 State Key Laboratory of Rice Biology and Breeding, China National Rice Research Institute, Hangzhou, PR China

**Keywords:** baculovirus, Ac16, nucleolar localization signal, IE1

## Abstract

*Autographa californica* multiple nucleopolyhedrovirus (AcMNPV) Ac16 (BV/ODV-E26) is a multifunctional protein found exclusively in group I NPV genomes. Here, we report a novel role for Ac16 in nucleolar localization. Using protein truncation and subcellular localization analysis, we characterized that the residues 78–113 of Ac16 contain nuclear (NLS) and nucleolar (NoLS) localization signals. Further multiple point mutation analysis within this region demonstrated that two basic-amino-acid-rich clusters, ^78^HKKKLRH^84^ and ^108^KKTTHR^113^, function as NLSs, and together, they constitute a functional NoLS. However, Ac16 itself was not observed to localize to nucleoli, while it displayed an overlapping distribution with IE1 during AcMNPV infection. Co-expression assay revealed that IE1, an Ac16-interacting protein, alters the subcellular localization of Ac16, and this effect is independent of the Ac16 NoLS. By yeast two-hybrid library screening, vacuolar (H^+^)-ATPase subunit D (SfVhaD) was identified as a candidate interaction partner of Ac16, which was further confirmed by co-immunoprecipitation. Ac16 affected the localization of SfVhaD; both proteins predominantly colocalized in nucleoli in transient co-expression assays, while they primarily colocalized within the virogenic stroma during AcMNPV infection. Together, these data suggest that Ac16 contains a functional NoLS and may facilitate the transport of its interaction partners from nucleoli to viral replication centres during AcMNPV infection.

## Introduction

Baculovirus, belonging to the family *Baculoviridae*, is a diverse group of large, circular, dsDNA viruses pathogenic to larvae of the insect orders Lepidoptera, Hymenoptera and Diptera [1]. The family is subdivided into four genera, including *Alphabaculovirus* (lepidopteran-specific NPV), *Betabaculovirus* (lepidopteran-specific GV), *Gammabaculovirus* (hymenopteran-specific NPV) and *Deltabaculovirus* (dipteran-specific NPV) [2,3]. The most studied baculovirus is *Autographa californica* multiple nucleopolyhedrovirus (AcMNPV), which belongs to the species *Alphabaculovirus aucalifornicae* within the genus *Alphabaculovirus*. Baculovirus replication occurs within the nucleus of the host cell, generating two morphologically distinct virion forms: budded viruses (BVs) during early infection and occlusion-derived viruses (ODV) during late infection. BVs mediate cell-to-cell spread within an infected host, while ODVs facilitate transmission between insect hosts [4,6].

AcMNPV Ac16 (also known as DA26 or BV/ODV-E26) and its homologues are multifunctional proteins exclusively found in Group I alphabaculoviruses [7]. Ac16 serves as a structural component in the envelopes of both BV and ODV and is able to associate with intracellular membranes via palmitoylation [8,9]. Beyond these roles, Ac16 participates in an extensive protein interaction network. Ac16 forms a complex with the viral protein FP25K and host actin [8]. Both Ac16 and FP25K are cross-linked to the viral inner nuclear membrane (INM)-sorting motif of ODV envelope proteins such as ODV-E66, demonstrating the involvement of Ac16 in INM-directed protein trafficking [10,11]. Additionally, Ac16 binds viral immediate-early proteins IE0 and IE1 [12]. This interaction is evolutionarily conserved. Bm8, the homologue of Ac16 in *Bombyx mori* nucleopolyhedrovirus (BmNPV), similarly interacts with IE1 and accumulates at nuclear foci during early infection [13,14]. Ac16 also associates with host ESCRT-III components (Ist1, Vps2A and Vps24) and viral core proteins, including Ac92, Ac93, P48 and ODV-E18 [15], while Bm8 interacts with multiple host proteins, including Serrate, Lipoprotein receptor, Laminin, Adf-1, Asperous and TANGO1 of *B. mori* [16].

Functional studies reveal species-specific phenotypes when Ac16 homologues are disrupted. AcMNPV lacking *ac16* replicates normally in cultured cells but exhibits somewhat enhanced larval infectivity [12,17]. In contrast, disruption of *Bm8* reduces BmNPV replication efficiency in *B. mori* cells, yet it expands the range of infectable tissues and shortens the time to lethality in larvae [13,18]. These findings reveal sophisticated adaptation mechanisms that modulate Ac16 function across different host environments.

The nucleolus, traditionally known as the site of ribosome biogenesis, is a dynamic subnuclear compartment involved in numerous cellular processes, including transcriptional regulation, cell signalling and cellular stress responses [19,20]. Given its various functions, the nucleolus serves as a key interface in virus–host interactions, with numerous viruses from diverse families targeting the nucleolus to hijack host functions for replication [21]. This targeting is often mediated by specific nucleolar localization signals (NoLSs) within viral proteins [22].

In this study, we identified a conserved NoLS spanning residues 78–113 of AcMNPV Ac16. Unexpectedly, Ac16 failed to accumulate in nucleoli during infection despite containing this functional signal. We further demonstrated that viral protein IE1 redirects the subcellular localization of Ac16 through a NoLS-independent mechanism. In addition, host protein vacuolar (H^+^)-ATPase subunit D (SfVhaD) was characterized as an Ac16 binding partner, with both proteins colocalizing in viral replication centres during infection.

## Methods

### Cell culture

*Spodoptera frugiperda* clonal isolate 9 (Sf9) cells were cultured at 27°C in Grace’s insect medium supplemented with 10% FBS (Invitrogen, USA), penicillin (25 unit ml^−1^) and streptomycin (25 µg ml^−1^).

### Construction of transient expression plasmids

A HA tag fusion expression plasmid, pBlue-ie1-HA-SV40, was constructed previously [23]. Primers are available in Table S2 (available in the online Supplementary Material). ORF of *ac16* and *Sfvhac* was amplified by using primers 1/2 and 59/60, respectively, and cloned into XbaI/PstI sites of pBlue-ie1-HA-SV40 to generate plasmids pBlue-Ac16:HA and pBlue-SfVhac:HA.

The coding sequences of HA tag in the plasmid pBlue-ie1-HA-SV40 were replaced by that of a FLAG tag (DYKDDDDK) using primers 3–6 to create pBlue-ie1-FLAG-SV40, according to the site-directed, ligase-independent mutagenesis (SLIM) strategy [24,25]. AcMNPV IE1 ORF was amplified by primers 7/8 and cloned into XbaI/PstI sites of pBlue-ie1-FLAG-SV40, creating plasmid pBlue-IE1:FLAG. Similarly, the SfVhaD ORF was amplified by primers 9/10 and cloned into XbaI/BamHI sites of pBlue-ie1-FLAG-SV40, producing pBlue-SfVhaD:FLAG.

### Construction of N/C-terminus truncated AcMNPV Ac16

A series of truncations was performed at the AcMNPV Ac16 N/C-terminus. The AcMNPV Ac16 ORF was truncated by segment deletion using the SLIM strategy. The plasmid pBlue-Ac16:HA was used as the target for mutagenesis, and each site-directed reaction included four primers. For N-terminal truncation of Ac16, primers 11/12 were used in all reactions, and along with primers 13/14 for the deletion of aa 2–77, primers 15/16 for the deletion of aa 2–89 and primers 17/18 for the deletion of aa 2–113, resulting in pBlue-Ac16_Δ2-77_:HA, pBlue-Ac16_Δ2-89_:HA and pBlue-Ac16_Δ2-113_:HA, respectively. To truncate Ac16 C-terminus, primer pairs 19/20 were used in all reactions, and along with primers 21/22 for the deletion of aa 147–225, primers 23/24 for the deletion of aa 114–225, primers 25/26 for the deletion of aa 95–225 and primers 27/28 for the deletion of aa 78–225, resulting in plasmids pBlue-Ac16_Δ147-225_:HA, pBlue-Ac16_Δ114-225_:HA, pBlue-Ac16_Δ95-225_:HA and pBlue-Ac16_Δ78-225_:HA, respectively. To express a mCherry-fused peptide encompassing Ac16 NoLS, the AcMNPV *ie-1* promoter and Ac16 aa 78–113 were PCR amplified from pBlue-Ac16_Δ2-77_:HA using primers 29/30, and *mcherry* with simian virus 40 (SV40) poly(A) was obtained from pBlue-NS:mCherry [26] using primers 31/32. These resulting PCR products were fused by a PCR-based DNA-assembly procedure, named double-joint PCR [27], using primers 29/32, and were inserted into SacI/XhoI sites of pBluescript II SK (+) vector, creating plasmid pBlue-Ac16^78-113^:mCherry.

### Multiple alanine point substitutions in AcMNPV Ac16

Multiple alanine point substitutions in Ac16 were generated by SLIM using plasmid pBlue-Ac16:HA as the template for mutagenesis. Basic-amino-acid-rich clusters in Ac16 NoLS of pBlue-Ac16:HA were substituted to alanines by using primers 33/34 for the mutation of BR1 (78-HKKKLRH-84, underlines denote mutation positions), primers 35/36 for the mutation of BR2 (90-RKK-92), primers 37/38 for the mutation of BR3 (101-RK-102) and primers 39/40 for the mutation of BR4 (108-KKTTHR-113), generating plasmids pBlue-Ac16^MutBR1^:HA, pBlue-Ac16^MutBR2^:HA, pBlue-Ac16^MutBR3^:HA and pBlue-Ac16^MutBR4^:HA. The plasmid pBlue-Ac16^MutBR1^:HA was used as the template in combination with primers 39/40 to create the plasmid pBlue-Ac16^MutBR1/4^:HA.

### Construction of subcellullar localization plasmids

A 1.3 kb WT *ac16* fragment containing the entire Ac16 ORF under the control of its native promoter and an OpMNPV *ie2* poly(A) signal was PCR amplified from a previously constructed bacmid *ac16*KO-AC16 [12] by using primers 41/42; the resulting fragment was cloned into pGEM-T Easy vector to create pGEM-Ac16. An HA tag was inserted into the C terminus of Ac16 by using primers 43–46, creating pGEM-Ac16:HA. The plasmid pGEM-Ac16:HA was digested with XhoI/SacI and inserted into pFACT, creating pFACT-Ac16:HA. A NS and GFP co-expressing cassette was amplified from pBlue-NS:GFP with primers 47/48; the resulting fragment was inserted into the SalI site of pFACT-Ac16:HA to replace the original GFP expression cassette, creating pFACT-NS:GFP-Ac16:HA.

The native promoter and poly(A) signal of *SfvhaD* from *S. frugiperda* genome, along with the *SfvhaD* ORF from the *S. frugiperda* cDNA library and the *gfp* ORF, were PCR amplified using primers 49–56. These fragments were ligated into the XhoI-digested pBluescript II SK (+) vector via the ClonExpress Ultra one-step cloning kit (Vazyme). The resulting plasmid was digested with XhoI and subcloned into pFACT-Ac16:HA, generating pFACT-Ac16:HA-SfVhaD:GFP.

### Bacmid constructions

The AcMNPV *ac16* knockout bacmid (AcBac-*ac16*KO) was previously generated [12]. Plasmids pFACT-Ac16:HA, pFACT-NS:GFP-Ac16:HA and pFACT-Ac16:HA-SfVhaD:GFP were used to transpose fragments into the *polyhedrin* locus of AcBac-*ac16*KO, according to the Bac-to-Bac expression manual (Invitrogen) for the construction of Bac-Ac16^HA^, Bac-NS:GFP-Ac16^HA^ and Bac-Ac16^HA^-SfVhaD^GFP^, respectively.

### Transfection

Bacmid DNA was purified from 500 ml LB cultures using a HiPure plasmid midiprep kit (Invitrogen), with concentrations quantified by NanoDrop 2000c spectrophotometer (Thermo Scientific). Sf9 cells were seeded into culture plates with an approximate density of 1×10^6^ cells per well and transfected with 2 µg bacmid DNA by using Cellfectin (Invitrogen) according to the instructions provided by the manufacturer. After 5-h incubation with transfection mixture, cells were replenished with fresh Grace’s medium.

### Confocal microscopy

Sf9 cells (5×10^5^) were seeded into 35 mm glass bottom dishes (*In Vitro* Scientific) to attach for 5 h. Monolayers were fixed in 4% paraformaldehyde for 15 min, washed three times with PBS, permeabilized for 15 min with PBS containing 0.3% Triton X-100 and blocked for 1 h with 2% BSA in PBS. Fixed and blocked cells were incubated overnight at 4°C with a mouse monoclonal anti-FLAG antibody (1:300; Abmart) or a rabbit polyclonal anti-HA antibody (1:100; Beyotime). After three 10-min washes in 2% BSA blocking buffer, cells were incubated for 1 h with an Alexa 488-conjugated goat anti-mouse IgG (1:300) or an Alexa 594-conjugated goat anti-rabbit IgG (1:300) (Invitrogen), followed by a 15-min nuclear stain with Hoechst 33342 (10 µg ml^−1^). Cells were washed with PBS and imaged using a Zeiss LSM 700 confocal microscope with ZEN 2011 software (Zeiss).

### Yeast two-hybrid assays

Yeast two-hybrid (Y2H) screening followed the Matchmaker Gold Yeast Two-Hybrid System User Manual (Clontech). The *ac16* ORF was amplified by primers 57/58 and cloned into EcoRI/BamHI sites of pGBKT7 to generate the bait plasmid pGBKT7-Ac16, expressing a fusion protein with the GAL4 DNA-binding domain. A cDNA library prepared from AcMNPV (E2 strain)-infected Sf9 cells was cloned into pGADT7 as the prey vector to express fusion proteins with the GAL4 activation domain. The Y2H gold yeast strain was cotransformed with the pGBKT7-Ac16 bait and the pGADT7-based cDNA library. Interaction confirmation was performed on QDO medium lacking tryptophan, leucine, histidine and adenine (SD/−Trp-Leu-His-Ade) supplemented with X-*α*-gal and aureobasidin A. Positive clones were sequenced and verified through one-to-one cotransformation of bait and prey plasmids into the Y2H Gold yeast strain.

### Co-immunoprecipitation

Sf9 cells were cotransfected with the plasmids expressing SfVhaD:FLAG and GFP-tagged Ac16 (4 µg of each plasmid). At 36 hpt, cells were collected and lysed for 10 min at 4°C in cell lysis buffer for Western blotting and IP (Beyotime) supplemented with a protein inhibitor cocktail (Roche). Debris was removed by centrifugation, and the supernatant was collected. For immunoprecipitation, the lysate supernatant was incubated overnight at 4°C with anti-FLAG M2 magnetic beads (Sigma) under rotation. Beads were washed five times with TBS (50 mM Tris-HCl, 150 mM NaCl, pH 7.4). Bound proteins were eluted using 3× FLAG peptide and then boiled in 1% SDS and *β*-mercaptoethanol. Proteins were analysed by SDS-PAGE and Western blotting.

## Results

### Identification of Ac16 as a nucleolus-associated protein

**Fig. 1.**
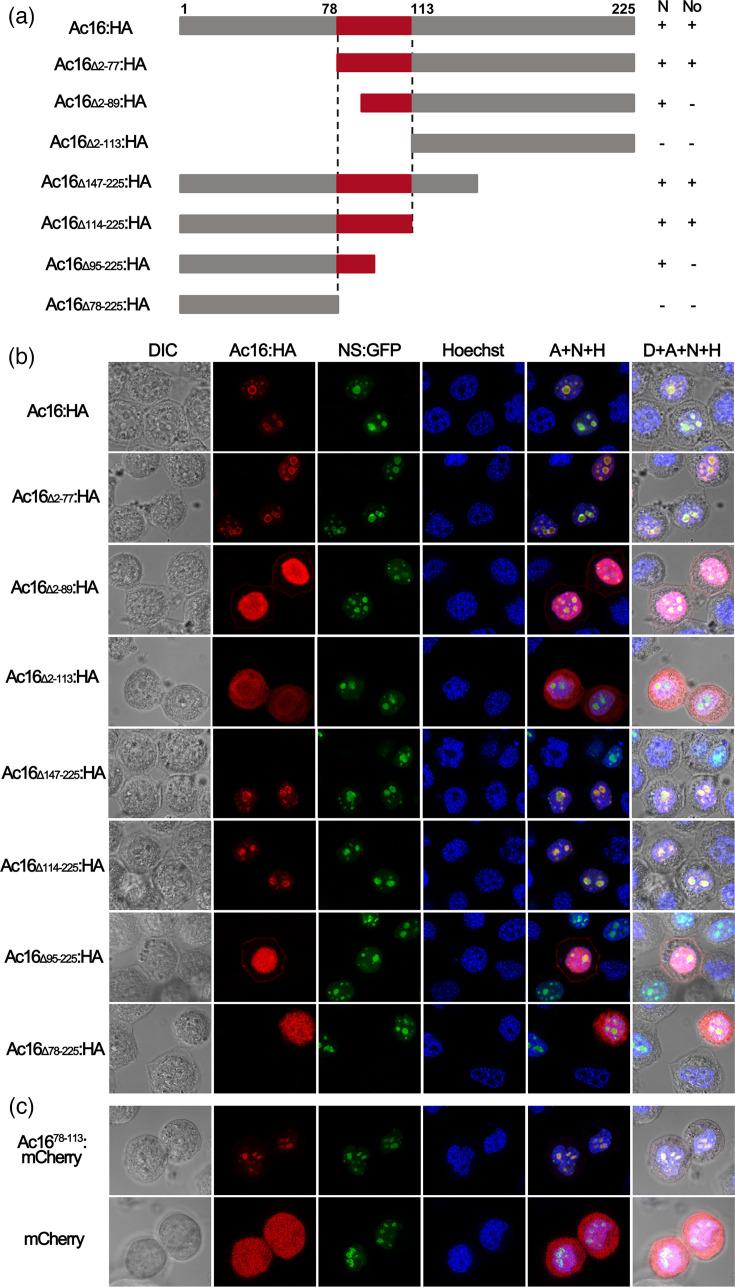
AcMNPV Ac16 truncations and localization analysis. (**a**) Schematic diagram depicting the N/C-terminal truncation of AcMNPV Ac16. Sequential truncations of AcMNPV Ac16 N-terminus (pBlue-Ac16_Δ2-77_:HA, pBlue-Ac16_Δ2-89_:HA and pBlue-Ac16_Δ2-113_:HA) and C-terminus (pBlue-Ac16_Δ147-225_:HA, pBlue-Ac16_Δ114-225_:HA, pBlue-Ac16_Δ95-225_:HA and pBlue-Ac16_Δ78-225_:HA) are carried out and named according to the amino acid residues deleted in Ac16. Nuclear (N) and/or nucleolar (No) localization of the indicated proteins was determined by immunofluorescence microscopy. A plus (+) in the N/No column indicates a strong nuclear/nucleolar association. (**b**) Subcellular localization analysis of AcMNPV Ac16 with N/C-terminal truncations. Sf9 cells were cotransfected with pBlue-NS:GFP and one of plasmids pBlue-Ac16_Δ2-77_:HA, pBlue-Ac16_Δ2-89_:HA, pBlue-Ac16_Δ2-113_:HA, pBlue-Ac16_Δ147-225_:HA, pBlue-Ac16_Δ114-225_:HA, pBlue-Ac16_Δ95-225_:HA or pBlue-Ac16_Δ78-225_:HA. Localization of full-length or truncated Ac16 was detected via Alexa fluor 594 labelled (red) HA tags. (**c**) Localization analysis of Ac16 NoLS. Sf9 cells were cotransfected with plasmids pBlue-Ac16^78-113^:mCherry and pBlue-NS:GFP. The subcellular localization was examined at 24 hpt. Red (Ac16), green (NS:GFP) and blue (Hoechst) fluorescence images were merged in the column labelled A+N+H, and light (DIC), red, green and blue fluorescence images were merged in the column labelled D+A+N+H. Images show representative cells from the entire population.

AcMNPV infection resulted in the redistribution of the nucleolus of infected Sf9 cells, and late expression factor 5 (LEF5) of most baculoviruses is found to contain at least one NoLS, which mediates its nucleolar localization and retention [26]. To identify additional nucleolus-associated proteins encoded by AcMNPV, we screened viral proteins with clusters of basic amino acids, a hallmark feature of NoLSs. This screening identified viral protein Ac16 as a candidate nucleolus-associated protein. The Ac16 ORF was thus cloned into a pBluescript transient expression vector, generating plasmid pBlue-Ac16:HA, where Ac16 was expressed in fusion with a HA tag under control of the AcMNPV *ie-1* promoter and SV40 poly(A) signal. For nucleolar visualization, we employed the previously constructed plasmid pBlue-NS:GFP, which expresses the Sf9 nucleolar protein Nucleostemin (NS) fused to GFP [26]. Sf9 cells were cotransfected with pBlue-Ac16:HA and pBlue-NS:GFP. Ac16 was monitored by immunofluorescence using a mouse monoclonal anti-HA antibody conjugated with the red Alexa Fluor 594 goat anti-mouse antibody, and NS was observed by green GFP fluorescence. By 24 h post-transfection (hpt), red immunofluorescence of Ac16 displayed a dot-like distribution within the nucleus, overlapping with the nucleolus labelled by NS:GFP (Fig. 1b). These results indicated that Ac16 localizes primarily to the nucleolus in Sf9 cells.

### Bioinformatic prediction and localization domain mapping

Homologues of AcMNPV Ac16 are found exclusively in lepidopteran Group I NPVs. We screened Ac16 homologues for potential NoLSs using the NoD predictor (http://www.compbio.dundee.ac.uk/www-nod/). Bioinformatic analysis revealed that 12 Group I alphabaculoviruses contain a single predicted NoLS in the central region of their Ac16 proteins, while prediction scores for the remaining homologues, including AcMNPV Ac16, approached the positive threshold (Table S1). These data suggested that NoLS is conserved in baculovirus Ac16s. To identify the functional nucleotides in AcMNPV Ac16, we generated a series of Ac16 mutants with N/C-terminal truncations in pBlue-Ac16:HA, generating plasmids pBlue-Ac16_Δ2-77_:HA, pBlue-Ac16_Δ2-89_:HA and pBlue-Ac16_Δ2-113_:HA, pBlue-Ac16_Δ147-225_:HA, pBlue-Ac16_Δ114-225_:HA, pBlue-Ac16_Δ95-225_:HA and pBlue-Ac16_Δ78-225_:HA (Fig. 1a). Each of the constructs was cotransfected with pBlue-NS:GFP into Sf9 cells and examined at 24 hpt. For Ac16 with N-terminal truncations, the deletion of residues 2–77 did not impair nuclear/nucleolar localization, whereas deletion to residue 89 (Ac16_Δ2-89_:HA) abolished nucleolar retention despite maintaining nuclear localization. Further truncation to residue 113 (Ac16_Δ2-113_:HA) resulted in an even distribution in both cytoplasm and nucleus of Sf9 cells (Fig. 1b). In turn to C-terminal truncations, abolishment of nucleolar localization was monitored by a further deletion of residues 95–113 from Ac16_Δ114-225_:HA, and a diffuse distribution throughout the cell was observed for Ac16_Δ78-225_:HA (Fig. 1b). Collectively, our results identified residues 78–113 as essential for both nuclear and nucleolar localizations of Ac16. To test whether this 36-amino-acid sequence alone mediates nucleolar targeting, we constructed mCherry-fused peptide encompassing Ac16 residues 78–113, creating the expression plasmid pBlue-Ac16^78-113^:mCherry. Transfection of Sf9 cells with plasmid pBlue-Ac16^78-113^:mCherry demonstrated pronounced nucleolar accumulation of the fusion protein by 24 hpt (Fig. 1c), confirming that Ac16 residues 78–113 function as an NoLS.

### Functional characterization of basic residue clusters within the NoLS

**Fig. 2.**
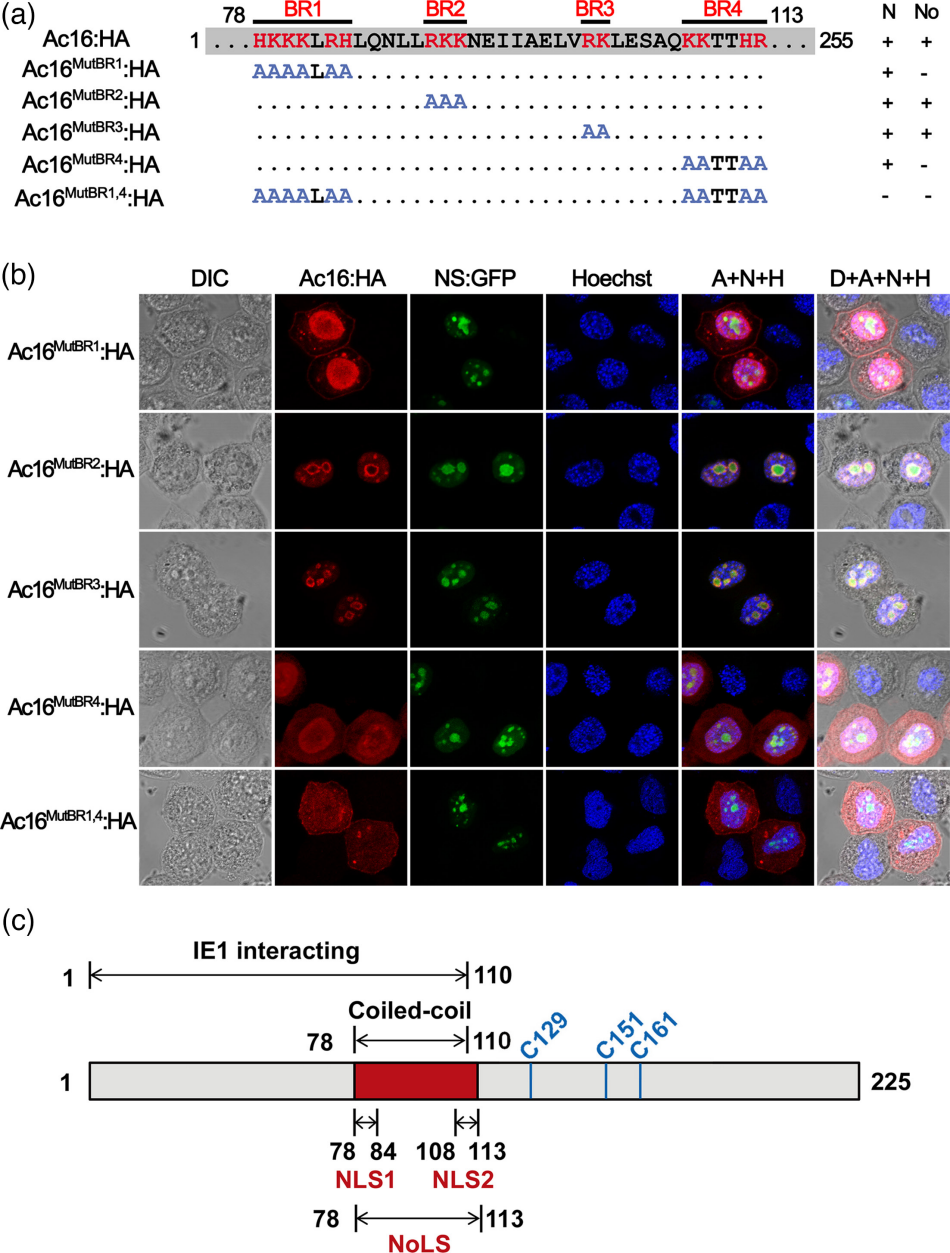
Mutational analysis of AcMNPV Ac16 NoLS. (**a**) Schematic diagram of mutation sites in AcMNPV Ac16. BR clusters of Ac16 were substituted by alanines as indicated in pBlue-Ac16:HA, creating plasmids pBlue-Ac16^MutBR1^:HA, pBlue-Ac16^MutBR2^:HA, pBlue-Ac16^MutBR3^:HA, pBlue-Ac16^MutBR4^:HA and pBlue-Ac16^MutBR1/4^:HA. Nuclear (N) and/or nucleolar (No) localization of indicated proteins was determined by immunofluorescence microscopy. A plus (+) in the N/No column indicates a strong nuclear/nucleolar association. (**b**) Subcellular localization analysis of Ac16 mutants. Sf9 cells were cotransfected with pBlue-NS:GFP and one of the plasmids pBlue-Ac16^MutBR1^:HA, pBlue-Ac16^MutBR2^:HA, pBlue-Ac16^MutBR3^:HA, pBlue-Ac16^MutBR4^:HA or pBlue-Ac16^MutBR1/4^:HA. Localization of wild-type or mutated Ac16 was monitored through Alexa fluor 594 labelled (red) HA tags at 24 hpt. Red (Ac16:HA), green (NS:GFP) and blue (Hoechst) fluorescence images were merged in the column labelled A+N+H, and light (DIC), red, green and blue fluorescence images were merged in the column labelled D+A+N+H. (**c**) Domain and motif composition of AcMNPV Ac16. Ac16 harbours two functional NLSs, ^78^HKKKLRH^84^ (NLS1) and ^108^KKTTHR^113^ (NLS2), which together constitute an NoLS (residues 78–113). The IE1-interacting domain was previously mapped to the N-terminal region (residues 1–110), which contains a putative coiled-coil domain (residues 78–110) [14]. Three cysteine residues (C129, C151 and C161) within Ac16 were predicted to serve as substrates for palmitoylation [9].

The peptide 78–113 of Ac16 contains four basic-amino-acid-rich (BR) clusters, including BR1 (^78^HKKKLRH^84^), BR2 (^90^RKK^92^), BR3 (^101^RK^102^) and BR4 (^108^KKTTHR^113^) (Fig. 2a). To investigate the functional role of these basic amino acids in the nuclear and nucleolar localization of Ac16, we generated multiple point mutations within each BR cluster in the expression vector pBlue-Ac16:HA, creating plasmids pBlue-Ac16^MutBR1^:HA, pBlue-Ac16^MutBR2^:HA, pBlue-Ac16^MutBR3^:HA and pBlue-Ac16^MutBR4^:HA (Fig. 2a). These plasmids were individually cotransfected with pBlue-NS:GFP into Sf9 cells. Using confocal fluorescence microscopy, red immunofluorescence was observed in the nucleus with strong accumulation in NS-positive nucleolus for pBlue-Ac16^MutBR2^:HA and pBlue-Ac16^MutBR3^:HA by 24 hpt (Fig. 2b), indicating BR2 and BR3 clusters are not required for nuclear/nucleolar localization of Ac16. However, nuclear localization without nucleolar association was observed for pBlue-Ac16^MutBR1^:HA and pBlue-Ac16^MutBR4^:HA (Fig. 2b), suggesting that both BR1 and BR4 clusters are essential for Ac16 nucleolar targeting. Plasmid pBlue-Ac16^MutBR1/4^:HA was further generated with combined mutations in both BR1 and BR4. When pBlue-Ac16^MutBR1/4^:HA was transfected into Sf9 cells, red immunofluorescence was found to distribute throughout the cell (Fig. 2b). These results demonstrated that both BR1 and BR4 stretches of AcMNPV Ac16 contain functional NLSs, and they cooperatively mediate nucleolar targeting (Fig. 2c).

### Colocalization of Ac16 and IE1 during infection

**Fig. 3.**
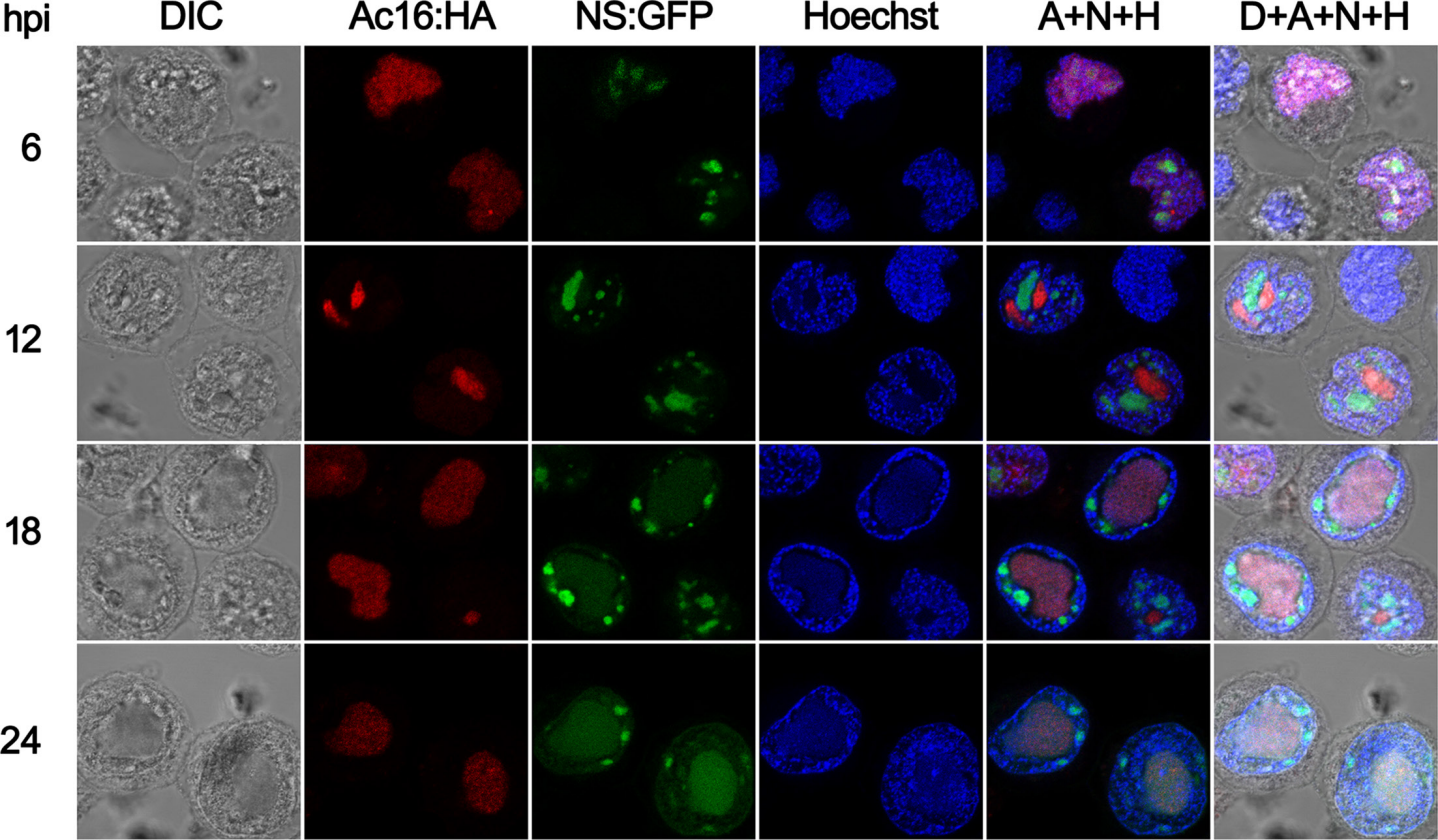
Ac16 localization analysis during AcMNPV infection. Sf9 cells were infected with Bac-NS:GFP-Ac16^HA^ at an m.o.i. of 5. At the indicated hours post-infection, cells were incubated with mouse monoclonal anti-HA antibody followed by Alexa Fluor 594-conjugated goat anti-mouse antibody for the detection of HA-tagged Ac16. Photographs were taken by using light microscopy (DIC), red immunofluorescence (Ac16:HA), GFP fluorescence microscopy (NS:GFP) or blue nuclear staining (Hoechst). Red, green and blue fluorescence images were merged in the column labelled A+N+H, and light, red, green and blue fluorescence images were merged in the column labelled D+A+N+H.

To visualize AcMNPV Ac16 distribution during infection, the HA-tagged *ac16* gene under the control of its native promoter and poly(A) signal was cloned into the *polyhedrin* locus of *ac16*-null bacmid [12], creating Bac-NS:GFP-Ac16^HA^. The *polyhedrin* and *ns:gfp* expression cassettes were also introduced by transposition into the same locus. Sf9 cells were infected with Bac-NS:GFP-Ac16^HA^ at an m.o.i. of 5. Confocal fluorescence microscopy showed that red immunofluorescence for Ac16 was evenly distributed in the nucleus at 6 hpi, while Ac16 formed discrete nuclear foci that did not overlap with NS:GFP-labelled nucleoli at 12 hpi (Fig. 3). A colocalization between Ac16 and NS:GFP was monitored in the centre of the nucleoplasm at 18 and 24 hpi (Fig. 3). The difference in Ac16 localization to the nucleolus in transient transfections but not in AcMNPV-infected cells suggested a viral factor was responsible for preventing or subverting Ac16 nucleolar localization.

**Fig. 4.**
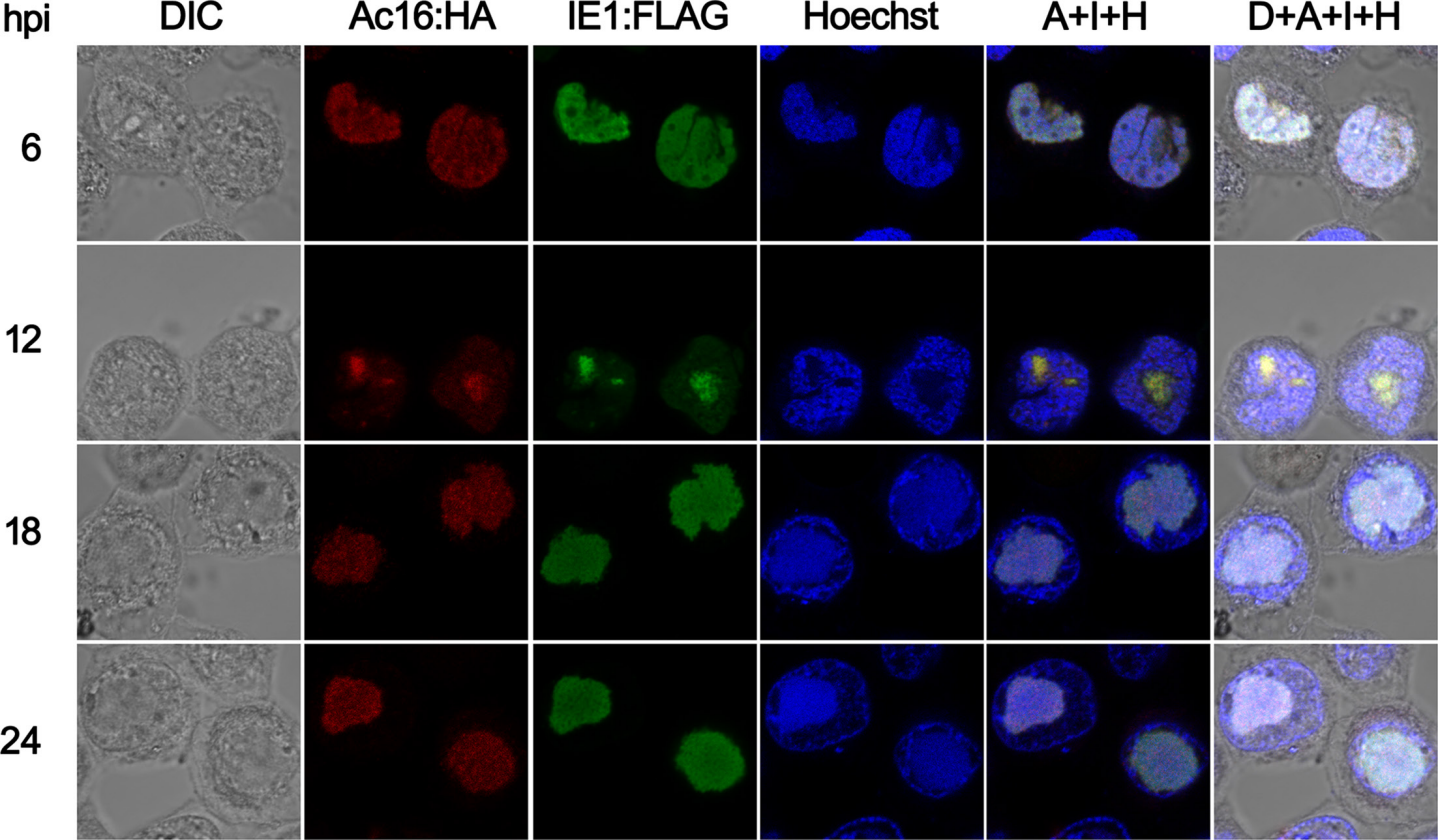
Colocalization analysis of Ac16 with IE1 during AcMNPV infection. Sf9 cells were transfected with pBlue-IE1:FLAG and followed by infection with Bac-Ac16^HA^ at an m.o.i. of 5. At the indicated hours post-infection, Ac16 was monitored through Alexa Fluor 594 labelled (red) HA tags, while IE1 was detected by Alexa Fluor 488 labelled (green) FLAG tags. Photographs were taken by using light microscopy (DIC), red immunofluorescence (Ac16:HA), GFP immunofluorescence (IE1:FLAG) or blue nuclear staining (Hoechst). Red, green and blue fluorescence images were merged in the column labelled A+I+H, and light, red, green and blue fluorescence images were merged in the column labelled D+A+I+H.

It was previously reported that Ac16 interacts with IE1, which localizes to the viral replication compartment, virogenic stroma (VS), during infection [12,28]. To determine whether Ac16 and IE1 are colocalized in AcMNPV-infected cells, the plasmid pBlue-IE1:FLAG was constructed by expressing IE1 fused with a FLAG tag under control of the AcMNPV *ie-1* promoter and SV40 poly(A) signal. Sf9 cells were transfected with pBlue-IE1:FLAG and followed by infection with an m.o.i. of 5 of Bac-Ac16^HA^, which was generated by inserting HA-tagged *ac16* and *polyhedrin* expression cassettes into the *ac16*-null bacmid. Colocalization of Ac16 and IE1 was observed from 6 to 24 hpi (Fig. 4). Our results demonstrated that Ac16 associates with IE1 during AcMNPV infection.

### NoLS-independent redirection of Ac16 by IE1

**Fig. 5.**
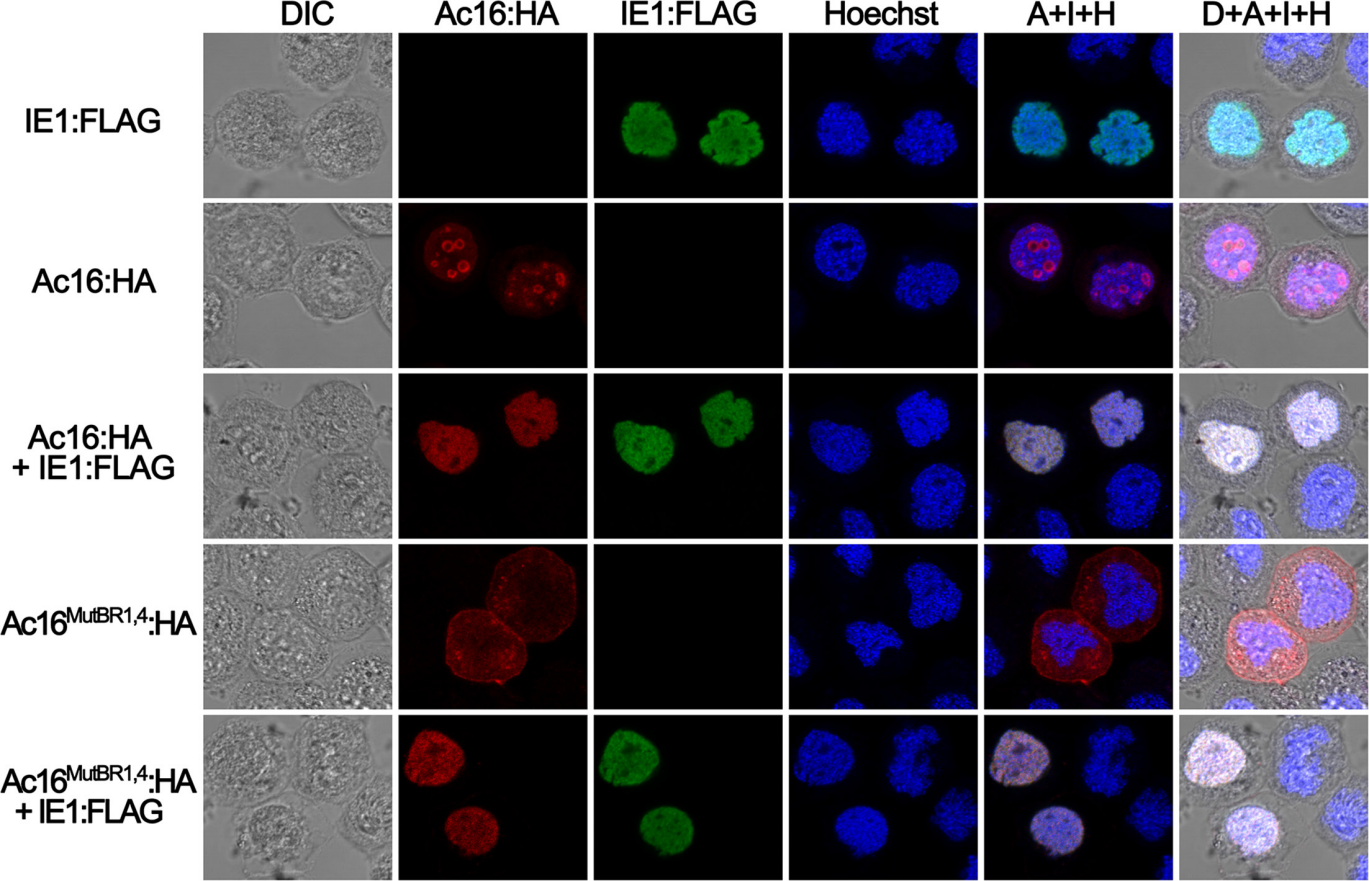
Localization analysis of Ac16 and NoLS-null Ac16^MutBR1/4^ in the presence or absence of IE1. Sf9 cells were transfected with the following plasmids: pBlue-IE1:FLAG, pBlue-Ac16:HA, pBlue-Ac16:HA/pBlue-IE1:FLAG, pBlue-Ac16^MutBR1/4^:HA or pBlue-Ac16^MutBR1/4^:HA/pBlue-IE1:FLAG. At 24 hpt, wild-type or NoLS-null mutant Ac16 was monitored through Alexa Fluor 594 labelled (red) HA tags, while IE1 was detected by Alexa Fluor 488 labelled (green) FLAG tags. Red (Ac16:HA), green (IE1:FLAG) and blue (Hoechst) fluorescence images were merged in the column labelled A+I+H, and light (DIC), red, green and blue fluorescence images were merged in the column labelled D+A+I+H.

We next examined whether IE1 alone alters Ac16 subcellular localization in the absence of AcMNPV infection. Sf9 cells were cotransfected with plasmids pBlue-Ac16:HA and pBlue-IE1:FLAG. The expression of Ac16 and IE1 was monitored by immunostaining with antibodies against HA (red) and FLAG (green), respectively. In transient transfections, full-length Ac16 was found exclusively in the nucleolus in the absence of IE1 (Fig. 5, row 2), whereas in the presence of IE1, Ac16 showed a pan-nuclear distribution and directly colocalized with IE1 (Fig. 5, row 3). These data demonstrated that IE1 could be the factor excluding Ac16 from nucleolar association.

BmNPV ORF8 (Bm8) is a homologue of AcMNPV Ac16. Residues 78–110 of Bm8 are required for its interaction with IE1 [14]. This region corresponds exactly to the NoLS motif in Ac16 (Fig. 2c). To investigate whether the Ac16 NoLS is also required for the interaction between Ac16 and IE1, plasmids pBlue-Ac16^MutBR1/4^:HA (expressing an Ac16 mutant lacking the NoLS) and pBlue-IE1:FLAG were cotransfected into Sf9 cells. The subcellular localization of Ac16^MutBR1/4^ shifted from an even cellular distribution in the absence of IE1 (Fig. 5, row 4) to nuclear retention in its presence (Fig. 5, row 5). This indicated that the BR1 and BR4 domains needed for nucleolar localization of Ac16 in transient infections were not involved in its interaction with IE1 and that IE1 was sufficient for nucleolar localization of Ac16.

### SfVhaD is a host interaction partner of Ac16 with nucleolar localization capacity

**Fig. 6.**
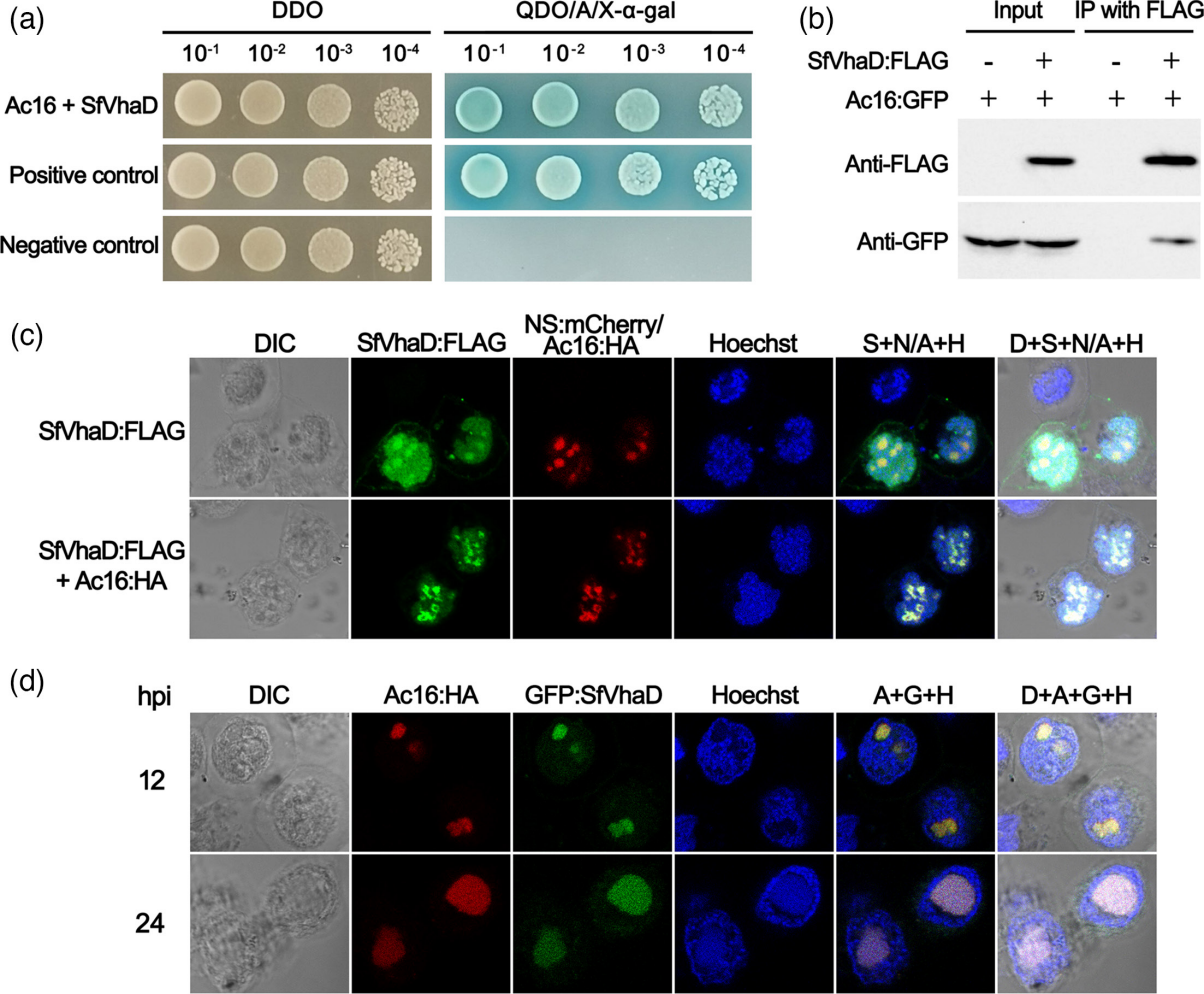
Interaction between Ac16 and SfVhaD. (**a**) The Y2H gold yeast strain was cotransformed with the plasmid pairs pGBKT7-Ac16 and pGADT7-SfVhaD. Plasmids pGBKT7-53 and pGADT7-T were cotransformed as positive controls, while pGBKT7-Lam and pGADT7-T served as negative controls. Transformant growth was verified on nonselective DDO plates (SD/-Leu/-Trp medium). Protein–protein interactions were assessed via reporter gene activation on selective QDO/A/X-*α*-gal medium (SD/-Ade/-His/-Leu/-Trp containing aureobasidin A and X-*α*-gal). (**b**) Sf9 cells were transfected with pBlue-SfVhaD:FLAG and pBlue-Ac16:GFP. At 36 hpt, cells were lysed and proteins immunoprecipitated using anti-FLAG antibody-conjugated beads. Samples were separated by 12% SDS-PAGE and analysed by Western blotting. Input lanes contained 1% of the total cell lysate and 5% of the immunoprecipitation eluate. Interactions were detected by immunoblotting precipitated proteins with anti-GFP antibody. (**c**) Sf9 cells were transfected with plasmid pairs pBlue-SfVhaD:FLAG/pBlue-NS:mCherry or pBlue-SfVhaD:FLAG/pBlue-Ac16:HA. At 24 hpt, SfVhaD was visualized using Alexa fluor 488 labelled (green) FLAG tags, while Ac16 was detected via Alexa fluor 594 labelled (red) HA tags. Green (SfVhaD:FLAG), red (NS:mCherry/Ac16:HA) and blue (Hoechst) fluorescence images were merged in the column labelled S+N/A+H, and light (DIC), red, green and blue fluorescence images were merged in the column labelled D+S+N/A+H. (**d**) Sf9 cells were infected with Bac-Ac16^HA^-SfVhaD^GFP^ at an m.o.i. of 5. At the indicated hours post-infection, cells were treated with mouse monoclonal anti-HA antibody followed by Alexa Fluor 594-conjugated goat anti-mouse antibody for the detection of HA-tagged Ac16. Photographs were taken by using light microscopy (DIC), red immunofluorescence (Ac16:HA), GFP fluorescence microscopy (GFP:SfVhaD) or blue nuclear staining (Hoechst). Red, green and blue fluorescence images were merged in the column labelled A+G+H, and light, red, green and blue fluorescence images were merged in the column labelled D+A+G+H.

We next sought to identify host or viral proteins interacting with AcMNPV Ac16. The full-length Ac16 coding sequences were inserted into the pGBKT7 vector to generate pGBKT7-Ac16, which served as bait in the Y2H screening against a cDNA library derived from wild-type AcMNPV (E2 strain) infected Sf9 cells. Initial screening identified the *S. frugiperda* homologue of vacuolar (H^+^)-ATPase (V-ATPase) subunit D, SfVhaD (GenBank accession number GHKU01108063.1), as a putative Ac16 interactor. To confirm the specificity of the interaction between Ac16 and SfVhaD, coding sequences of SfVhaD were cloned into pGADT7 to create plasmid pGADT7-SfVhaD for one-to-one hybridization. The Y2H gold yeast strain was cotransformed with pGBKT7-Ac16 and pGADT7-SfVhaD. Plasmids pGBKT7-53 and pGADT7-T were cotransformed as positive controls, while pGBKT7-Lam and pGADT7-T were cotransformed as negative controls. The Y2H assays showed that yeast cells cotransformed with pGBKT7-Ac16/pGADT7-SfVhaD grew as well as the positive controls on QDO/A/x-*α*-gal medium, while no sign of growth was detected in the negative controls (Fig. 6a). Subsequently, co-immunoprecipitation (Co-IP) assays were performed to validate the interaction between Ac16 and SfVhaD. FLAG tag and GFP were fused to the N- or C-terminus of SfVhaD and Ac16, generating plasmid pBlue-SfVhaD:FLAG and pBlue-Ac16:GFP, respectively. The resultant plasmids were cotransfected into Sf9 cells. At 36 hpt, cells were lysed and subjected to immunoprecipitation using anti-FLAG antibody-conjugated beads. Western blot analysis of the immunoprecipitates using an anti-GFP antibody revealed clear bands corresponding to Ac16:GFP (Fig. 6b), confirming a specific interaction between Ac16 and SfVhaD.

### Ac16 and SfVhaD colocalize in viral replication centres during infection

SfVhaD is evolutionarily conserved across diverse eukaryotes, exhibiting protein identities ranging from 43.6% to 93.5% to its homologues (Table S3). To monitor the subcellular localization of SfVhaD, Sf9 cells were cotransfected with pBlue-SfVhaD:FLAG and pBlue-NS:mCherry, which was constructed previously and expressed mCherry-tagged NS [26]. Immunofluorescence analysis revealed predominant nuclear and nucleolar localization of SfVhaD, with partial enrichment in cytoplasmic (likely endocytic) compartments (Fig. 6c), where it colocalized with the highly conserved V-ATPase subunit c (SfVhac) in *S. frugiperda* (Fig. S1, Table S3). As Ac16 interacts with SfVhaD, their localization might be influenced by each other. We thus transfected Sf9 cells with plasmids pBlue-Ac16:HA and pBlue-SfVhaD:FLAG. The intracellular localization of SfVhaD and Ac16 was observed by mouse anti-FLAG antibody-conjugated green and rabbit anti-HA antibody-conjugated red immunofluorescence, respectively. In the absence of Ac16:HA, SfVhaD:FLAG localized primarily to both the nucleus and nucleolus (Fig. 6c, row 1). However, in the presence of Ac16:HA, SfVhaD:FLAG showed enriched nucleolar localization along with Ac16:HA (Fig. 6c, row 2), suggesting Ac16 affected the localization of SfVhaD in Sf9 cells. To further investigate subcellular distribution in the presence of infection, HA-tagged Ac16 and GFP-tagged SfVhaD under the control of their native promoters were introduced into the *ac16*-null bacmid, generating Bac-Ac16^HA^-SfVhaD^GFP^. Sf9 cells were infected with Bac-Ac16^HA^-SfVhaD^GFP^ at an m.o.i. of 5, and the results demonstrated that Ac16 and SfVhaD primarily colocalized within the VS during AcMNPV infection (Fig. 6d).

## Discussion

Growing evidence indicates that viruses from diverse families sequester host nucleolar proteins or target viral proteins to the nucleolus to facilitate viral replication [22,29]. Consistent with this pattern, our previous study demonstrated that AcMNPV infection triggers nucleolar disassembly and redistribution. We further identified that the LEF5 contains a NoLS, and mutation of this signal impaired progeny production and occlusion body formation [26]. Expanding on these findings, this study revealed that Ac16, a multifunctional AcMNPV protein, also localizes to the nucleolus but not during an actual infection.

Although most nucleolar proteins possess basic residue-rich regions that function as NoLSs, a precise consensus sequence for NoLSs has not been defined [30]. Through subcellular localization analysis of Ac16 N- and C-terminal truncation mutants, we identified a BR spanning residues 78–113 as the functional NoLS (Fig. 1c). Since nucleolar proteins must first traverse the nuclear envelope, they often harbour both NLS and NoLS, whose sequences may overlap [21]. NS1 protein of influenza A H3N2 subtype virus possesses a C-terminal NLS that also functions as a NoLS and targets the protein into the nucleolus [31]. The NLS (residues 32–56) of human 58 kDa microspherule protein (MSP58) also functions as a NoLS [32]. In this study, bioinformatic analysis showed that the residues 78–113 of Ac16 contain four clusters of basic amino acids that share features characteristic of NoLSs. Further truncation and mutation analysis within this region demonstrated that BR1 (^78^HKKKLRH^84^) and BR4 (^108^KKTTHR^113^) clusters function as NLSs, and together they constitute a functional NoLS. Thus, our results demonstrated that residues 78–113 function as an NoLS.

To our surprise, despite harbouring this functional NoLS, Ac16 was not observed to localize to nucleoli during AcMNPV infection (Fig. 3). Instead, it displayed an overlapping distribution pattern with IE1 (Fig. 4). Co-expression assays demonstrated that IE1 alone was sufficient to redirect subcellular localization Ac16 (Fig. 5), indicating that IE1 plays a dominant role in determining Ac16 trafficking. However, although IE1 is a viral immediate-early expressed protein, we cannot exclude the possibility that other viral factors participate in Ac16 movement, given the multiple interacting partners previously reported for Ac16 [8,15].

The mechanism underlying NoLS-mediated protein targeting to the nucleolus is not fully elucidated. Unlike nuclear import, the nucleolus is a membrane-free subnuclear body, which does not require active transport machinery for nucleolar delivery [33]. Nucleolar targeting of proteins has been assumed to be mediated via direct or indirect interactions with constitutive nucleolar components, such as resident proteins, rRNA or rDNA [34]. Our data indicate that while Ac16 possesses intrinsic nucleolar localization capability, its subcellular trafficking during infection is predominantly mediated by IE1 and coincides with VS development. Notably, mutations in the Ac16 NoLS did not disrupt the Ac16-IE1 interaction (Fig. 5). Therefore, we hypothesize that the primary role of Ac16 in nucleolar localization is not its own retention, but rather to facilitate the relocalization of nucleolar components (which interact with Ac16) away from the nucleolus to the viral replication site, the VS. In support of this hypothesis, V-ATPase subunit D (SfVhaD) is a nuclear and nucleolar protein and was identified as an interacting partner of Ac16 by Y2H screening (Fig. 6a, c). Co-IP and colocalization experiments confirmed that Ac16 interacts with SfVhaD, and both proteins accumulate within the VS during infection (Fig. 6b–d).

V-ATPases are large, multisubunit complexes composed of a peripheral V_1_ domain responsible for ATP hydrolysis and an integral V_0_ domain that mediates proton translocation across membranes [35]. These proton pumps are ubiquitously present in various endomembrane systems and plasma membranes and are essential for pH regulation of the intracellular compartments, the cytoplasm and the extracellular space [36]. V-ATPases are also reported to localize to the nuclear envelope, where they generate H^+^ gradients and regulate nuclear pH [37], consistent with our observation of SfVhac localization to the nuclear membrane (Fig. S1). Importantly, V-ATPase activity contributes to antiviral defence. It is a key component of the silkworm defence response against BmNPV [38]. Supporting this role, both the expression level and activity of V-ATPase are significantly higher in BmNPV-resistant silkworm strains compared to susceptible ones [38,39]. Furthermore, overexpression of the V-ATPase 16 kDa subunit (a component of the V_0_ domain) in BmNPV-infected silkworm cells could significantly inhibit BmNPV proliferation [38]. Among V-ATPase subunits, subunit D (VhaD) has been identified as a critical regulator governing the coupling efficiency between proton transport by V_0_ and ATP hydrolysis by V_1_ [40,41]. Building upon this knowledge of the antiviral function and regulatory mechanisms of V-ATPase, our results demonstrated that AcMNPV may utilize Ac16 to interact with and restrain the function of SfVhaD within the VS. We propose that this interaction disrupts the normal coupling and activity of the host V-ATPase complex, maintaining it in a state of low activity. This viral strategy is likely employed to counteract the host V-ATPase-mediated antiviral defence, thereby promoting efficient viral infection. However, deletion of *ac16* does not disturb AcMNPV replication in cultured cells; it even slightly enhances viral infectivity in larvae [12,17]. These results suggested that while the Ac16-SfVhaD interaction may modulate host V-ATPase activity during infection, its precise role in viral pathogenesis remains unclear. To fully elucidate the biological relevance of this host-virus interaction, further studies are required to dissect the regulatory dynamics and functional consequences of V-ATPase activity during AcMNPV infection.

In summary, we identified a functional NoLS at amino acids 78–113 of AcMNPV Ac16, and the NoLS is well conserved in baculovirus Ac16s. However, Ac16 does not associate with the nucleolus during early infection. Instead, IE1 mediates the subcellular mobilization of Ac16, which is not dependent on Ac16 NoLS. SfVhaD, V-ATPase subunit D, is further characterized as an interaction partner of Ac16, and both proteins are colocalized in viral replication centre during AcMNPV infection.

## Supplementary material

10.1099/jgv.0.002246 Uncited Supplementary Material 1.

## Abbreviations

AcMNPV: *Autographa californica* multiple nucleopolyhedrovirus
BmNPV: *Bombyx mori* nucleopolyhedrovirus
BV: budded virus
Co-IP: co-immunoprecipitation
LEF5: late expression factor 5
NoLS: nucleolar localization signal
ODV: occlusion-derived virus
SLIM: site-directed, ligase-independent mutagenesis
VS: virogenic stroma
Y2H: yeast two-hybrid
