# Supplementary material for: Identification of the nucleolar localization signal in Autographa californica multiple nucleopolyhedrovirus multifunctional protein Ac16
Source: J Gen Virol. 2026 Apr 1;107(4):002246. doi: 10.1099/jgv.0.002246 (PMC13043108; doi:10.1099/jgv.0.002246)
Supplement: Uncited Supplementary Material 1. [file jgv-107-02246-s001.pdf]

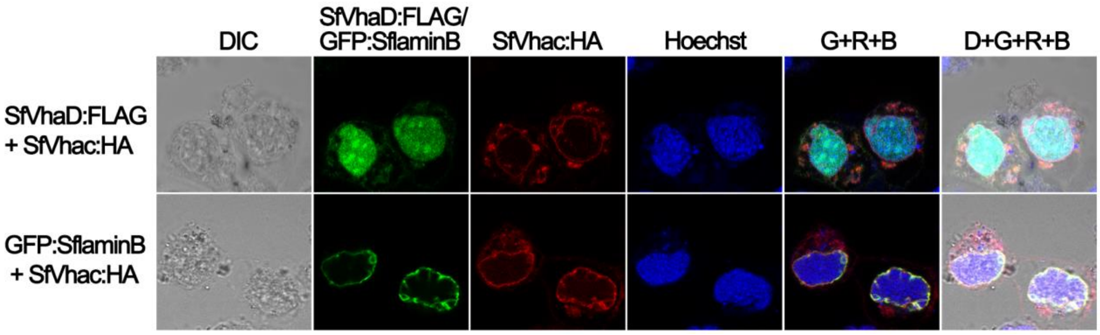

Supplementary Figure S1. Localization analysis of SfVhaD and SfVhac. Sf9 cells were transfected with plasmid pairs pBlue-SfVhaD:FLAG/pBlue-SfVhac:HA or pBlue-GFP:SflaminB/ pBlue-SfVhac:HA. The plasmid pBlue-GFP:SflaminB expresses GFP-fused SflaminB, a B-type lamin homolog in *S. frugiperda* [1]. At 24 hpt, SfVhaD was visualized using Alexa fluor 488 labelled (green) FLAG tags, while SfVhac was detected via Alexa fluor 594 labelled (red) HA tags. Green (SfVhaD:FLAG), red (NS:mCherry/Ac16:HA), and blue (Hoechst) fluorescence images were merged in the column labeled G+R+B, and light (DIC), red, green, and blue fluorescence images were merged in the column labeled D+G+R+B.

References

1. **Chen GQ, Li P, Yan Q, Wu YH, Wang HR et al.** Identification of Spodoptera frugiperda importin alphas that facilitate the nuclear import of Autographa californica multiple nucleopolyhedrovirus DNA polymerase. *Insect Molecular Biology* 2021;30(4):400-409.

Supplementary Table S1. Predicted NoLS in Ac16 homologs

**Name of Virus**	**NoLS or NoLS-like amino acid sequence**	**Protien size (aa)**	**Relative position**	**Accession no.**
Autographa californica MNPV (AcMNPV)	-	225	-	NP_054045.1
Antheraea pernyi NPV (AnpeNPV)	KTRICFNKLSRLQRKRVRNMQKLLRKKNTI	197	51-80	YP_611098.1
Anticarsia gemmatalis NPV (AngeNPV)	TQLQKRRVKNMQRLVRKKNNI	215	73-93	ALR71525.1
Bombyx mandarina NPV (BomaNPV)	-	229	-	ACQ57199.1
Bombyx mori NPV (BmNPV)	-	229	-	AIS92740.1
Catopsilia pomona NPV (CapoNPV)	-	281	-	YP_009255379.1
Choristoneura fumiferana DEF MNPV (CfDEFMNPV)	LTQLQKRRVKNMQKLVRKKNNI	215	72-93	NP_932624.1
Choristoneura fumiferana MNPV (CfMNPV)				
Choristoneura murinana NPV (ChmuNPV)	SQLQRKRVRNMQKLIRRKNGV	196	57-77	YP_008992227.1
Choristoneura occidentalis NPV (ChocNPV)	SQLQRKRVRNMQKLIRRKNSV	200	61-81	AGR57024.1
Choristoneura rosaceana MNPV (ChroMNPV)	LSQLQKKRLRNMQKLIRRKNSV	199	59-80	YP_008378490.1
Condylorrhiza vestigialis MNPV (CoveMNPV)	TQLQKRRVKNMQRLVRKKNT	215	73-92	YP_009118607.1
Cyclophragma undans NPV (CyunNPV)	-	244	-	AOT85593.1

Dasychira pudibunda NPV (DapuNPV)	LSQLQRRRVRNMQKLIRKKNSV	197	59-80	AKR14222.1
Dendrolimus kikuchii NPV (DekiNPV)	-	234	-	AFS52011.1
Epiphyas postvittana NPV (EppoNPV)	LTQLQKKRVRNMQRLVRKKNNI	204	62-83	NP_203182.1
Hyphantria cunea NPV (HycuNPV)	-	127	-	YP_473325.1
Lonomia obliqua MNPV (LoobMNPV)	-	240	-	AKN81077.1
Maruca vitrata MNPV (MaviMNPV)	-	209	-	YP_950738.1
Orgyia pseudotsugata MNPV (OpMNPV)	LSQLQRRRVRNMQKLIRKKNSV	197	59-80	NP_046171.1
Oxyplax ochracea NPV (OxocMNPV)	-	181	-	AVA31196.1
Philosamia cynthia ricini NPV (PhcyNPV)	KTRICFNKLSRLQRKRVRNMQKLLRKKNTI	197	51-80	AFY62931.1
Plutella xylostella MNPV (PlxyMNPV)	-	74	-	ABE68402.1
Rachiplusia ou MNPV (RaouMNPV)	-	225	-	AAN28087.1
Spilosoma obliqua NPV (SpobNPV)	SQLQRKRVRNMQRLIRKKNNV	196	60-80	AUR45154.1
Thysanoplusia orichalcea NPV (ThorNPV)	-	224	-	YP_007250427.1

Supplementary Table S2. Primers used in this study

Primer no. and name	Primer sequence (5'–3')	Position in genome
(1) Ac16-upX	GCTCTAGAATGGAGTCTGTTCAAACGCG	XbaI + AcMNPV (13092-13111)
(2) Ac16-dnP	AAACTGCAGATAGGCGTTAATATCACTTTGAGAT	PstI + AcMNPV (13742-13766)
(3) FLAG-FL	ATGGACTACAAAGACGATGACGATAAAGTCGAGTAAGAA TTCGATATCAAGCTTGGG	CDS of FLAG tag + pBluescript II SK (+) (689-706)
(4) FLAG-FS	TAAGAATTCGATATCAAGCTTGGG	pBluescript II SK (+) (689-706)
(5) FLAG-RL	CTCGACTTTATCGTCATCGTCTTTGTAGTCCATCTGCAGCC CGGGGGATCCAC	CDS of FLAG tag + pBluescript II SK (+) (707-726)
(6) FLAG-RS	CTGCAGCCCGGGGGATCCAC	pBluescript II SK (+) (707-726)
(7) IE1-upX	GCTCTAGAATGACGCAAATTAATTTTAACGC	XbaI + AcMNPV (127198-127220)
(8) IE1-dnP	AAACTGCAGATTAAATTCGAATTTTTTATATTTACAATTTA G	PstI + AcMNPV (128911-128943)
(9) SfVhaD-upX	GCTCTAGAATGTCTGGAAAAGATAGGCTAGC	XbaI + CDS of *SfvhaD* (1-23)
(10) SfVhaD-dnB	CGGGATCCGAACAGCAGGTCCTCGTCGC	BamHI + CDS of *SfvhaD* (719-738)
(11) D2RL	CATTCTAGAGCGGCCGCAGTCACTTGGTTG	Adaptor + AcMNPV (127185-127197)
(12) D2RS	CGGCCGCAGTCACTTGGTTG	AcMNPV (127185-127197)
(13) D2-77FL	CTCTAGAATGCATAAAAAGAAACTGCGGCAT	Adaptor + AcMNPV (13323-13343)
(14) D2-77FS	CATAAAAAGAAACTGCGGCAT	AcMNPV (13323-13343)
(15) D2-89FL	CTCTAGAATGAGAAAAAAAGAACGAAATTATTGCC	Adaptor + AcMNPV (13359-13382)
(16) D2-89FS	AGAAAAAAAGAACGAAATTATTGCC	AcMNPV (13359-13382)
(17) D2-113FL	CTCTAGAATGAATATTAGTAAACCAGCTCATTGG	Adaptor + AcMNPV (13431-13454)
(18) D2-113FS	AATATTAGTAAACCAGCTCATTGG	AcMNPV (13431-13454)
(19) D225FL	CTGCAGTATCCCTACGATGTTCCAGACTACGC	Adaptor + CDS of HA tag (5-26)
(20) D225FS	CCTACGATGTTCCAGACTACGC	CDS of HA tag (5-26)
(21) D147-225RL	GATACTGCAGACGTCTCCTTACAAACTTTTCG	Adaptor + AcMNPV (13508-13529)
(22) D147-225RS	ACGTCTCCTTACAAACTTTTCG	AcMNPV (13508-13529)
(23) D114-225RL	GATACTGCAGTCTGTGCGTTGTCTTCTTCTG	Adaptor + AcMNPV (13410-13430)
(24) D114-225RS	TCTGTGCGTTGTCTTCTTCTG	AcMNPV (13410-13430)
(25) D95-225RL	GATACTGCAGTTCGTTCTTTTTTCTTAGCAAATTTTG	Adaptor + AcMNPV (13347-13373)
(26) D95-225RS	TTCGTTCTTTTTTCTTAGCAAATTTTG	AcMNPV (13347-13373)
(27) D78-225RL	GATACTGCAGTACAGACTGTATCTTATTGAAATTCAAG	Adaptor + AcMNPV (13295-13322)
(28) D78-225RS	TACAGACTGTATCTTATTGAAATTCAAG	AcMNPV (13295-13322)
(29) Nols-up	CGAGCTCGCGAATGCAGCTGATC	SacI + AcMNPV (126881-126896)
(30) Nols-dn	CTCCTCGCCCTTGCTCACCATTCTGTGCGTTGTCTTCTTCT G	mCherry (1-21) + AcMNPV (13410-13430)
(31) mCherry-up	ATGGTGAGCAAGGGCGAGGAG	mCherry (1-21)
(32) mCherry-dn	CCCCTCGAGATCCAGACATGATAAGATACATTGATG	XhoI + Simian virus (2535–2561)
(33) Ac16mut1-F	GTAGCGGCGGCGGCGCTGGCGGCGTTGCAAAATTTGCTA AGAAAAAAGAACG	Ala substitution sequences for 78HKKKLRH84 + AcMNPV (13344-13371)
(34) Ac16mut1-R	CAACGCCGCCAGCGCCGCCGCCGCTACAGACTGTATCTTA TTGAAATTCAAGTGC	Ala substitution sequences for 78HKKKLRH84 + AcMNPV (13292-13322)
(35) Ac16mut2-F	CTAGCGGCGGCGAACGAAATTATTGCCGAGTTGGTT	Ala substitution sequences for

		90RKK92 + AcMNPV (13368-13391)
(36) Ac16mut2-R	GTTCGCCGCCGCTAGCAAATTTTGCAAATGCCGCAG	Ala substitution sequences for 90RKK92 + AcMNPV (13335-13358)
(37) Ac16mut3-F	GTTGCGGCGCTTGAAAGTGCACAGAAGAAGACAAC	Ala substitution sequences for 101RK102 + AcMNPV (13398-13423)
(38) Ac16mut3-R	AAGCGCCGCAACCAACTCGGCAATAATTTCGTTC	Ala substitution sequences for 101RK102 + AcMNPV (13367-13391)
(39) Ac16mut4-F	CAGGCGGCGACAACGGCGGCGAATATTAGTAAACCAGCTCATTGGAAATAC	Ala substitution sequences for 108KKTTHR113 + AcMNPV (13431-13460)
(40) Ac16mut4-R	ATTCGCCGCCGTTGTCGCCGCCTGTGCACTTTCAAGTTTTCTAACC	Ala substitution sequences for 108KKTTHR113 + AcMNPV (13388-13411)
(41) Ac16WT-upX	GCGCTCGAGCTACCTACAAAAAACACATGG	XbaI + AcMNPV (12714-12734)
(42) Ac16WT-dnS	GCGGAGCTCTTTGAGTGAGCATCGTTCCT	SacI + OpMNPV *ie2* polyA (164-185)
(43) Ac16H-FL	TATCCCTACGATGTTCCAGACTACGCTTAACTGCAGGGATCGATATCTG	CDS of HA tag + OpMNPV *ie2* polyA (1-22)
(44) Ac16H-FS	TAACTGCAGGGATCGATATCTG	OpMNPV *ie2* polyA (1-22)
(45) Ac16H-FL	AGCGTAGTCTGGAACATCGTAGGGATAATAGGCGTTAATATCACTTTGAGATTC	CDS of HA tag + AcMNPV (13740-13766)
(46) Ac16H-RS	ATAGGCGTTAATATCACTTTGAGATTC	AcMNPV (13740-13766)
(47) NS-upS	ACGCGTCGACCGCGAATGCAGCTGATCACG	SalI + AcMNPV (126880-126899)
(48) NS-dnS	ACGCGTCGACATCCAGACATGATAAGATACATTGATG	SalI + Simian virus (2535–2561)
(49) SfVhaDp-up	GGTACCGGGCCCCCCCTCGAGTTGATTCCCTGGTGGCTTTG	pBluescript II SK (+) (630-650) + SfVhaD promotor (1553-1572)
(50) SfVhaDp-dn	GCCTATCTTTTCCAGACATATTGATTTAGATAAATATTTGGCGTTTT	CDS of *SfvhaD* (1-19) + *SfvhaD* promotor (1-28)
(51) SfVhaD-up	TATGTCTGGAAAAGATAGGCTAGCG	CDS of *SfvhaD* (1-24)
(52) SfVhaD-dn	TTGCTCACCATGAACAGCAGGTCCTCGTCGC	CDS of *gfp* (1-11) + CDS of *SfvhaD* (719-738)
(53) GFP-up	CTGCTGTTCATGGTGAGCAAGGGCGAGG	CDS of *SfvhaD* (730-738) + CDS of *gfp* (1-19)
(54) GFP-dn	CTACTTGTACAGCTCGTCCATGCC	CDS of *gfp* (697-717)
(55) SfVhaDt-up	TGGACGAGCTGTACAAGTAGGCGCCGCGCCGATAT	CDS of *gfp* (701-717) + *SfvhaD* poly(A) (1-15)
(56) SfVhaDt-dn	ATCGATACCGTCGACCTCGAGAAACGCTAATTGTTCGTGATGTCC	pBluescript II SK (+) (625-645) + *SfvhaD* poly(A) (929-952)
(57) Ac16-upYEI	CCGGAATTCATGGAGTCTGTTCAAACGCG	EcoRI + CDS of *ac16* (1-20)
(58) Ac16-dnYB	CGGGATCCCATAGGCGTTAATATCACTTTGAGAT	BamHI + CDS of *ac16* (651-675)
(59) SfVhac-upX	GCTCTAGAATGGCCGAAAACCCAATCTAC	XbaI + CDS of *Sfvhac* (1-21)
(60) SfVhac-dnP	AAACTGCAGCTGTTTCGTGTAAAGGTAGATGG	PstI + CDS of *Sfvhac* (446-468)

Adapter: Complementary sequences for DNA fragment truncation

Supplementary Table S3. Sequence identity of V-ATPase subunits D and c across organisms

Organism	Subunit D (V_1)		Subunit c (V_0)		Reference
	Accession no.	Identity (%)	Accession no.	Identity (%)	
Spodoptera frugiperda	GHKU01108063.1	-	GHKU01039825.1	-	
Bombyx mori	NP_001040286.1	93.5	NP_001091762.1	94.6	[1, 2]
Drosophila melanogaster	NP_651987.1	85.4	NP_476801.1	90.4	[3]
			NP_729707.1	68.7	
			NP_001189086.1	83.2	
			NP_611169.1	65.9	
			XP_035458742.1	54.4	
Saccharomyces cerevisiae	NP_010863.1	48.7	NP_010887.3	69.2	[4]
Neurospora crassa	KHE84398.1	43.6	XP_961418.2	71.9	[5]
Schizosaccharomyces pombe	NP_588513.1	44.5	NP_594799.1	71.9	[6]
Arabidopsis thaliana	NP_191432.1	47.5	NP_179244.1	61.4	[7]
Oryza sativa	XP_015636169.1	50.2	NP_001410096.1	63.3	[7]
Mus musculus	NP_076210.1	74.8	NP_001348460.1	82.0	[4]
Homo sapiens	NP_057078.1	74.8	NP_001185498.1	79.6	[4]

Note: Percent identity values represent amino acid sequence similarity compared to the corresponding *S. frugiperda* subunit. Dash (-) indicates the reference sequence.

References

1. **Guo H, Huang C, Jiang L, Cheng T, Feng T et al.** Transcriptome analysis of the response of silkworm to drastic changes in ambient temperature. *Applied microbiology and biotechnology* 2018;102(23):10161-10170.

2. **Xie E, Guo H, Jiang L, Xia Q**. Identification of the Vo domain of V-ATPase in Bombyx mori silkworm. *International journal of biological macromolecules* 2020;163:386-392.

3. **Allan AK, Du J, Davies SA, Dow JA**. Genome-wide survey of V-ATPase genes in Drosophila reveals a conserved renal phenotype for lethal alleles. *Physiological genomics* 2005;22(2):128-138.

4. **Marshansky V, Rubinstein JL, Grüber G**. Eukaryotic V-ATPase: novel structural findings and functional insights. *Biochimica et biophysica acta* 2014;1837(6):857-879.

5. **Bowman EJ, Bowman BJ**. Cellular role of the V-ATPase in Neurospora crassa: analysis of mutants resistant to concanamycin or lacking the catalytic subunit A. *The Journal of experimental biology* 2000;203(Pt 1):97-106.

6. **Iwaki T, Goa T, Tanaka N, Takegawa K**. Characterization of Schizosaccharomyces pombe mutants defective in vacuolar acidification and protein sorting. *Molecular genetics and genomics : MGG* 2004;271(2):197-207.

7. **Schumacher K, Krebs M**. The V-ATPase: small cargo, large effects. *Current opinion in plant biology* 2010;13(6):724-730.
